# Assessment of safety and effectiveness after percutaneous closure for decannulation of Veno-Arterial Extracorporeal Membrane Oxygenation: A systematic review and meta-analysis

**DOI:** 10.1177/11297298241312753

**Published:** 2025-01-29

**Authors:** Tomás Marques Pereira, Diana Martins-Fernandes, Ana Rita Ferreira, Henrique Guedes da Rocha, Mario D’Oria, João Rocha-Neves

**Affiliations:** 1 Faculty of Medicine of University of Porto, Porto, Portugal; 2 Department of Intensive Care Medicine, Unidade Local de Saúde de São João, Porto, Portugal; 3 Department of Angiology and Vascular Surgery, Unidade Local de Saúde De Santo António, Porto, Portugal; 4 Division of Vascular Surgery, Cardiovascular Department, University Hospital of Trieste Azienda Sanitaria Universitaria Giuliano Isontina, Trieste, Italy; 5 Division of Vascular and Endovascular Surgery, Cardiovascular Department, University Hospital of Trieste Azienda Sanitaria Universitaria Giuliano Isontina, Trieste, Italy; 6 RISE@Health, Departamento de Biomedicina – Unidade de Anatomia, Faculdade de Medicina, Universidade do Porto, Porto, Portugal; 7 Department of Biomedicine – Unity of Anatomy, Faculty of Medicine of University of Porto, Porto, Portugal

**Keywords:** Percutaneous closure, mechanical circulatory support, limb ischemia

## Abstract

**Introduction:**

Veno-Arterial Extracorporeal Membrane Oxygenation (VA-ECMO) has emerged as a crucial component of critical care medicine, mainly as a lifesaving intervention for patients experiencing refractory cardiac arrest and respiratory failure.

**Background:**

In the past, VA-ECMO decannulation was surgical and often associated with a high rate of periprocedural complications, such as surgical site infection, bleeding, and patient mobilization costs. To reduce the rate of these adverse events, many percutaneous techniques utilizing suture-mediated closing devices have been adopted. One of those devices is the Perclose Proglide^®^ (PP).

**Objective:**

This study’s goal was to perform a systematic review to evaluate PP devices’ success and complication rates for VA-ECMO decannulation.

**Methods:**

To analyze the outcomes of PP in VA-ECMO decannulation, a systematic review of the most recent literature was conducted. The Medline, Web of Science, and Cochrane databases were systematically searched up to September 2023. The National Health, Blood, and Lung Institute Study quality assessment tools were used.

**Results:**

The final analysis included 10 observational studies comprising 418 patients. The efficacy of PP in VA-ECMO decannulation was 93.0% (95% CI 90.1%–96.0%). In 381 patients, the incidence of acute limb ischemia after VA-ECMO decannulation was 2.5% (95% CI 0.9%–4.%), the infection of the puncture site after decannulation was 1% (95% CI 0%–2%) in 385 patients. The incidence of patients with pseudoaneurysm after decannulation was 1.1% (95% CI 0.1%–2.1%).

**Conclusion:**

This systematic review and meta-analysis demonstrate the safety and efficacy of the PP for achieving hemostasis after VA-ECMO decannulation, with a high success rate and low rate of major complications.

## Introduction

Veno-Arterial Extracorporeal Membrane Oxygenation (VA-ECMO) has emerged as a crucial component of critical care medicine, mainly as a lifesaving intervention for patients experiencing refractory cardiac arrest and respiratory failure, helping maintain peripheral perfusion as well as restoring end-organ function.^1^ The use of ECMO as a cardiopulmonary support, easiness of implantation, increasing availability with the development of mobile ECMO teams, and a broadening range of indications have contributed to its expansion in the last years.^2^ Data from the Extracorporeal Life Support Organization (ELSO) Registry show a rise in ECMO utilization throughout the years, recording 16.803 in 2022.^3^

VA-ECMO is usually initiated through cannulas placed in the common femoral artery (CFA) and the common femoral vein (CFV), either with an open or percutaneous approach.^4^ Even though initially, VA-ECMO decannulation has been done with surgery, that practice may often be associated with high patient mobilization costs and a high rate of peri-procedural complications such as bleeding and site infection, with the latter being reported to be as high as 45% in a study done by Haddad et al.,^5^ and patient mobilization costs. Therefore, to reduce the rate of these adverse events, many percutaneous techniques utilizing suture-mediated closing devices have been adopted. One of those devices is the Perclose Proglide^®^ (PP; Abbott laboratories, released 2004, Perclose ProGlide™ Suture-Mediated Closure (SMC) System, Illinois, Chicago, USA).^6^

The PP is a device that actively approximates and sutures arteriotomy sites percutaneously. It operates by being inserted over a guidewire until blood flow indicates its correct placement within the lumen, then deploys “feet” to secure against the vessel wall, followed by needle deployment to create a suture loop that closes the arteriotomy upon tightening. Approved for closing 5–21 F arteriotomy sites as per its Instructions for Use (IFU), the PP requires two devices and a “preclose” technique for sheaths larger than 8 F.^7^

Despite the abundance of contemporary evidence on the safety and effectiveness of the PP for closing large-bore arterial access after endovascular aneurysm repair or transcatheter aortic valve implantation,^8,9^ much less is known about its utilization in ECMO. Therefore, this study aimed to perform a systematic review to evaluate the safety and effectiveness of using the PP device for percutaneous VA-ECMO decannulation.

## Methods

This systematic review was conducted following the Preferred Reporting Items for a Systematic Review and Meta-analysis (PRISMA) Statement and Assessing the methodological quality of systematic reviews (AMSTAR).^10,11^ Due to the nature of this study, an institutional review board’s ethical approval was not obtained. The review protocol has been registered on Prospero (reference: CRD42023478774).

The PICO framework for the study described might be outlined as follows: Population (P): Patients undergoing percutaneous Veno-Arterial Extracorporeal Membrane Oxygenation (VA-ECMO) decannulation. Intervention (I): Use of Perclose ProGlide (PP) device to close large-bore arterial access after endovascular aneurysm repair. Comparison (C): The PP device will be evaluated without a direct comparison due to the lack of head-to-head comparisons. Outcome (O): The PP device’s safety and effectiveness for percutaneous VA-ECMO decannulation.

### Selection criteria

Inclusion criteria included all original cohort or experimental studies performed in humans (except for case series under 10 patients) aged 18 years old and over, in which patients underwent percutaneous VA-ECMO arterial cannula removal with PP. No exclusion was made based on the publication language or publication date.

### Search strategy

A systematic search was performed in three databases (PubMed, Web of Science, and Cochrane) in September 2023. The query and keywords are shown in Supple-mental Table 1 and included terms such as “ECMO” OR “Extracorporeal membrane oxygenation” OR “Extracorporeal Circulation” and “ProGlide” OR “Perclose” OR “vascular closure devices.” The last search was conducted on September 30, 2023. Additionally, the references of the included primary studies and relevant available systematic reviews were screened to search for any further articles of possible interest.

### Study selection and data extraction

After duplicates were removed, two authors (TP and JRN) independently participated in study selection; any disagreement was solved by the intervention of a third author (ARF). First, studies were selected by title and abstract, and the remaining ones were eligible for full-text assessment. Efforts were made to contact the authors to obtain the full texts that were not publicly available.

Two authors (TP and JRN) independently extracted data from the included studies. Data was extracted using a .xls purposely built form on the year of publication, continent, recruitment center, study design, recruitment time, number of participants undergoing percutaneous VA-ECMO removal using PP, participants’ age and gender distribution, frequency of cardiovascular comorbidities, platelet number before decannulation, and use of antiplatelet and anticoagulation medication. In addition, data related to the technical success of PP, as well as the incidence of 30 days/short-term technical success and adverse events after VA-ECMO decannulation, was also retrieved.

### Assessment of study quality

Regarding qualitative assessment, the National Heart, Lung, and Blood Institute (NHLBI) Study Quality Assessment Tool was used for observational cohort and cross-sectional studies (2013).^12^ Two authors (TP and JRN) independently performed this assessment, and when disagreements were observed, decisions were made by mutual consensus after a third-party review (ARF).

### Quantitative synthesis

A random-effects meta-analysis (using the restricted maximum likelihood method) of log-transformed proportions was performed to calculate the meta-analytical pooled incidence of an efficient and safe percutaneous closure among participants. Pooled estimates and 95% confidence intervals (95% CI) were backtransformed into their original scale to simplify their interpretation. Heterogeneity was assessed using the Q-Cochran *p*-value and the *I*^2^ statistic—a *p*-value <0.10 and an *I*^2^ ⩾ 50% represent substantial heterogeneity. Sources of heterogeneity were assessed by leave-one-out sensitivity analysis. Assessed covariates included the publication year, participants’ mean age, percentage of male participants, percentage of patients with arterial atherosclerotic risk factors, and percentage of patients using antiplatelet and anticoagulant drugs.

All statistical analyses were performed using Open Meta^®^ (MetaMorph, Inc).

### Publication bias

To assess the potential for publication bias in the included studies, a funnel plot was constructed and visually inspected for asymmetry, which can indicate the presence of bias. Additionally, the Egger’s regression test was performed.

## Results

### Search results

**Figure 1.**
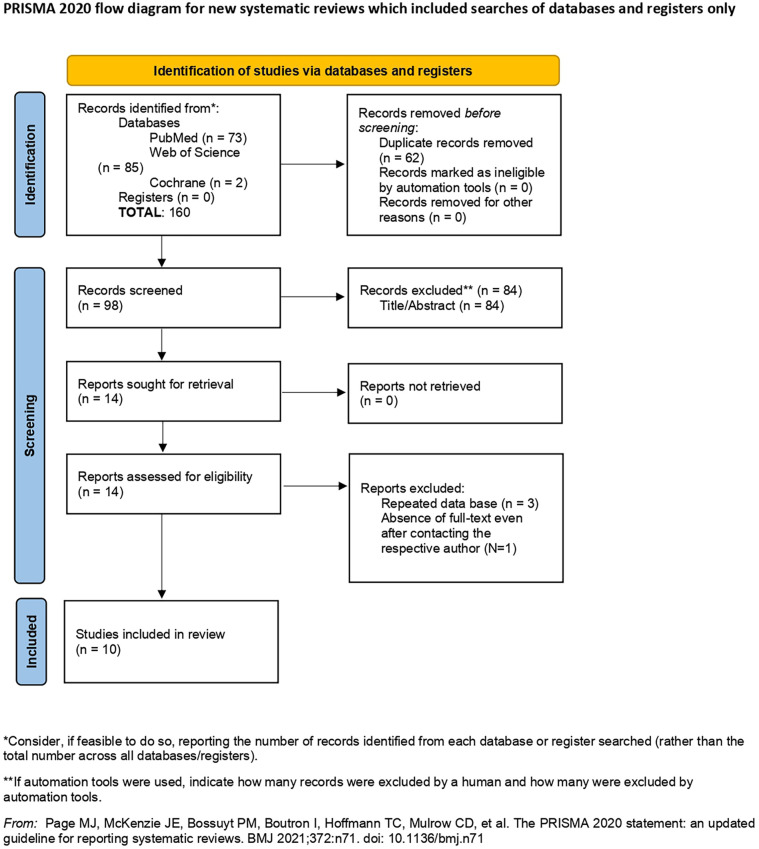
Flow-diagram according to PRISMA statement regarding the process of identification and selection of the studies.

After the database search and duplicate exclusion, 160 studies were screened. Upon selection by title and abstract, 86 studies were excluded. Fourteen studies were eligible for full-text assessment, and during this process, no studies were excluded or retrieved (Figure 1). Comprehensive reasons for exclusion upon full-text assessment were: repeated database (*N* = 3) and absence of full-text even after contacting the respective author (*N* = 1). Thus, a total of 10 published articles were included in this systematic review and included in the meta-analysis (Table 1).

**Table 1.** Characteristics of the studies included in the systematic revision.

Author	Publication year	Journal	Study center	Study design	Number of patients
Sun et al.^13^	2023	*Perfusion*	Xiamen Cardiovascular Hospital of Xiamen University, School of Medicine	Retrospective Cohort	30
Au et al.^17^	2022	*Artificial Organs*	Queen Elizabeth Hospital, Kowloon, Hong Kong	Retrospective Cohort	44
Hwang et al.^14^	2016	*Journal of Vascular Surgery*	Samsung Medical Center, Sungkyunkwan University School of Medicine	Retrospective Cohort	56
Chandel et al.^15^	2021	*JTCVS Techniques*	Inova Fairfax Hospital	Retrospective Cohort	51
Scherer et al.^16^	2023	*Frontiers in Cardiovascular Medicine*	Ludwig-Maximilians-University	Retrospective Cohort	33
Liu et al.^18^	2021	*Frontiers in Medicine*	The Second Affiliated Hospital of Zhejiang University School of Medicine, Hangzhou	Retrospective Cohort	24
Majunke et al.^20^	2016	*The Journal of Invasive Cardiology*	University of Leipzig, Heart Center, Department of Internal Medicine/Cardiology, Leipzig, Germany	Retrospective Cohort	15
Martin-Tuffreau et al.^19^	2021	*JTCVS Techniques*	Henri-Mondor University Hospital (Créteil France)	Prospective Case Series	22
Hayakawa N et al^22^	2023	Annals of vascular surgery	Multicenter	Retrospective Case series	37
Pellenc Q et al^21^	2020	European Journal of Cardio-Thoracic Surgery	Bichat Hospital, Paris University, Paris, France	Retrospective Cohort	106

### Description of studies

This review includes eight observational cohort studies and two case series. Overall, nine of those studies were retrospective,^13–21^ and one was prospective.^22^ Characteristics of the studies and case series can be found in Table 1. The included publications were performed in seven countries within three continents (one from North America, four from European countries, and five from Asia). A total of 418 patients were assessed, from a minimum of 15 up to a maximum of 106 patients per study. The included studies reported eight punctures of the common femoral artery and vein under ultrasound guidance.^13,15–17,19–22^ The mean age of the participants was 56.2 years. The percentage of male participants was 56.0% (*n* = 248). Demographics and comorbidities of the populations included in the studies were gathered and are available in Table 2, while Table 3 reports procedure-related variables. Data related to cannula removal adverse events, such as surgical open repair for cannula removal, acute limb ischemia, wound infection, arterial thrombosis, pseudoaneurysm, arterial-venous fistula, hematoma, arterial dissection, and procedure-related death are displayed in Table 4.

**Table 2.** Summary of clinical data of patients included.

Author	Mean age	Male gender (%)	BMI	HT (%)	DLD (%)	DM (%)	Smoking (%)	CAD (%)	CKD (%)	AFib (%)	Mean PlatBD	Anticoagulation therapy (%)	Antiplatelet therapy (%)
Sun et al.^13^	65.5	22 (73.3)	28.8	16 (53.3)	N/A	9 (30)	N/A	12 (40)	5 (16.7)	2 (6.7)	N/A	Warfarin – 2 (10)	ASA – 25 (83.3) DAPT – 24 (80)
Au et al.^17^	54.5	33 (75)	N/A	N/A	N/A	N/A	N/A	N/A	N/A	N/A	N/A	N/A	N/A
Hwang et al.^14^	53.3	19 (33.9)	23.4	18 (32.1)	N/A	19 (33.9)	9 (16.1)	N/A	4 (7.1)	5 (8.9)	112.09	Heparin – 48 (85.7) Warfarin – 1 (1.8)	ASA – 24 (42.9) DAPT – 23 (41.1)
Chandel et al.^15^	56	33 (64.7)	27.1	22 (43.1)	N/A	22 (43.1)	N/A	23 (45.1)	4 (7.8)	N/A	107	N/A	N/A
Scherer et al.^16^	58.6	24 (72.7)	27.3	N/A	N/A	N/A	N/A	N/A	N/A	N/A	77	Heparin – 32 (97.0)	ASA – 23 (69.7) Prasugrel – 15 (45.5) Clopidogrel – 2 (6.1) Ticagrelor – 2 (6.1)
Liu et al.^18^	42	10 (41.7)	24.3	4 (16.7)	2 (8.3)	2 (8.3)	3 (12.5)	1 (4.2)	N/A	N/A	N/A	19 (79.2)	6 (25)
Majunke et al.^20^	61	11 (73.3)	25.8	9 (60)	7 (46.7)	7 (46.7)	6 (40)	N/A	N/A	N/A	N/A	N/A	N/A
Martin-Tuffreau et al.^19^	60	N/A	N/A	N/A	N/A	N/A	N/A	N/A	N/A	N/A	N/A	N/A	N/A
Hayakawa et al.^22^	65.4	21 (56.8)	23.1	16 (43.2)	14 (37.8)	12 (32.4)	17 (45.9)	17 (45.9)	15 (40.5)	5 (13.5)	N/A	Heparin –14 (37.8) DOAC – 4 (13.5) Warfarin – 1 (2.7)	ASA – 19 (51.1) Prasugrel – 17 (45.9) DAPT – 16 (43.2) Ticlopidine – 1 (2.7)
Pellenc et al.^21^	53.5	75 (70.8)	23.9	27 (25.5)	N/A	15 (14.2)	69 (65.1)	14 (13.2)	N/A	N/A	N/A	N/A	N/A

AFib: atrial fibrillation; ASA: acetylsalicylic acid; BMI: body mass index; CAD: coronary artery disease; CKD: chronic kidney disease; DAPT: dual antiplatelet therapy (ASA + P2Y12 Inhibitor); DLD: dyslipidemia; DM: diabetes mellitus; DOAC: direct oral anticoagulant; HT: arterial hypertension; N/A: unavailable data; PlatBD: platelet number before decannulation.

**Table 3.** Procedure related variables.

Author	Indication for VA-ECMO – number of patients (%)	Puncture site	Cannula size – number (%)	Number of PP used per patient – number of patients (%)	PP used per patient (mean)	Use of preclose technique	Use of fluroscopy	Time spent in ECMO (days)	ICU instay (days)	Hospital instay (days)	PP efficacy (%)
Sun et al.^13^	Acute coronary syndrome – 22 (73.3) Fulminant myocarditis – 4 (13.3) Severe aortic stenosis – 3 (10) Pulmonary Embolism – 1 (3.3)	CFA	15F – 21 (70) 17F – 9 (30)	1 – 2 (6.7) 2 – 21 (70) 3 – 7 (23.3)	N/A	No	No	2.3	N/A	19.5	30 (100)
Au et al.^17^	Acute myocardial infarction – 20 (45.5) Shock post open heart operation – 14 (31.8) Pulmonary embolism – 4 (9.1) Myocarditis – 2 (4.5) Post cardiac catheter-based procedure complications – 2 (4.5) Refractory arrhythmia – 1 (2.3) Drug overdose – 1 (2.3)	CFA	15F – 21 (47.7) 17F – 22 (50) 19F – 1 (42.3)	2 – 24 (54.5) 3 – 18 (40.9) 4 – 1 (2.3) 5 – 1 (2.3)	N/A	No	No	4.2	N/A	N/A	38 (86.4)
Hwang et al.^14^	Cardiogenic shock – 44 (78.6) Septic shock – 7 (12.5) Respiratory failure – 4 (7.1) Hypovolemic shock – 1 (1.8)	CFA	14F – 2 (3.6) 15F – 4 (7.1) 16F – 35 (62.5) 17F – 13 (23.2) 18F – 2 (2.1)	N/A	N/A	No	No	N/A	23.8	43.51	48 (85.7)
Chandel et al.^15^	Left heart failure – 44 (86.3) Right heart failure – 5 (9.8) Acute pulmonary embolism – 3 (5.9)	CFA	17F^*a*^	N/A	N/A	Yes	Yes	6	8	N/A	46 (90.2)
Scherer et al.^16^	STEMI – 14 (42.4) NSTEMI – 10 (30.3) Decompensated CMP – 5 (15.1) Myocarditis – 3 (9.1) Other – 1 (3.0)	PFA	15F – 11 (33.3) 17F – 21 (63.6) 19F – 1 (3.0)	N/A	2	No	No	3.9	N/A	N/A	29 (87.9)
Liu et al.^18^	N/A	CFA	<18F – 6 (25) >18F – 18 (75)	N/A	2.1	No	No	N/A	N/A	13.7	23 (95.8)
Majunke et al.^20^	Refractory cardiogenic shock – 15 (100)	CFA	17F – 2 (13) 18F – 3 (20) 19F – 12 (67)	N/A	N/A	No	No	7.2	N/A	32	15 (100)
Martin-Tuffreau et al.^19^	N/A	CFA	17–19F	N/A	N/A	Yes	Yes	6	N/A	N/A	20/22 (90.9)
Hayakawa et al.^22^	Acute myocardial infarction–related conditions – 17 (45.9) Other – 12 (32.4) Other ventricular fibrillation/ventricular tachycardia – 8 (21.6)	CFA	16.5F^*b*^	N/A	N/A	No	Yes	3.2	21	N/A	35 (94.6)
Pellenc et al.^21^	Interstitial Lung disease – 69 (65.1) Obstructive Lung disease – 34 (32.1) Others – 3 (2.8)	CFA	15F – 106 (100)	N/A	N/A	Yes	No	0.77	N/A	N/A	98 (92.5)

CFA: common femoral artery; CMP: cardiomyopathy; F: French; NSTEMI: non-ST-elevation myocardial infarction; STEMI: ST-elevation myocardial infarction; PCI: percutaneous coronary intervention; PFA: proximal femoral artery; PP: Perclose Proglide.

a Median.

b Mean.

**Table 4.** Short-term outcomes and adverse events.

Author	Open repair following PP device failure (%)	Acute limb ischemia (%)	Arterial thrombosis (%)	Pseudoaneurysm (%)	Wound infection (%)	Arterious venous fistula (%)	Hematoma (%)	Arterial dissection (%)	Procedure related death (%)
Sun et al.^13^	0 (0)	3 (10)	2 (6.7)	2 (6.7)	1 (3.3)	1 (3.3)^*a*^	3 (10)	N/A	N/A
Au et al.^17^	6 (13.6)	0 (0)	2 (4.5)	2 (4.5)	1 (2.3)	0 (0)	N/A	2 (4.5)	0 (0)
Hwang et al.^14^	1 (1.8)	2 (3.6)	N/A	1 (1.8)	0 (0)	N/A	N/A	N/A	N/A
Chandel et al.^15^	4 (7.8)	2 (3.9)	N/A	0 (0)	1 (2.0)	N/A	N/A	N/A	0 (0)
Scherer et al.^16^	2 (6.1)	2 (6.1)	N/A	1 (3.0)	N/A	N/A	N/A	N/A	N/A
Liu et al.^18^	1 (4.2)	0 (0)	0 (0)	1 (4.2)	0	0	1 (4.2)	0 (0)	N/A
Majunke et al.^20^	2 (13.3)	2 (13.3)	N/A	N/A	2 (13.3)	N/A	N/A	1 (6.7)	0 (0)
Martin-Tuffreau et al.^19^	2 (9.1)	0 (0)	0 (0)	0 (0)	1 (4.5)	0 (0)	0 (0)	N/A	N/A
Hayakawa et al.^22^	0 (0)	N/A	N/A	1 (2.7)	0 (0)	0 (0)	2 (5.4)	N/A	0 (0)
Pellenc et al.^21^	3 (2.8)	2 (1.9)	5 (4.7)	0 (0)	0 (0)	N/A	N/A	1 (0.9)	N/A

N/A: unavailable data; PP: Perclose Proglide.

a There is lack of evidence to fully determine that this event was caused by decannulation or the puncture process.

### Main findings and meta-analysis

**Figure 2.**
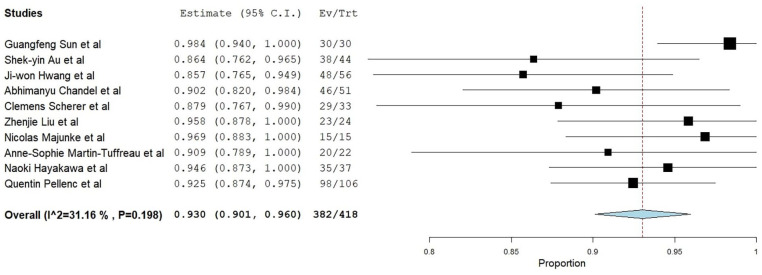
Forest plot of the meta-analytical technical success rate of percutaneous puncture (PP).

In 418 patients, PP’s meta-analytical technical success rate in VA-ECMO decannulation was 93.0% (95% CI 90.1%–96.0%, Standard error (SE) 1.2%, *p* < 0.001) *I*^2^ = 31.6% (Figure 2). In all results of leave-one-out sensitivity, the overall estimate remained stable across all iterations.

Additionally, subgroup analysis regarding the use of the pre-closure technique revealed an efficiency similar to the post-removal technique (91.7% CI: 87.7%–95.9%, SE 2.1%, *p* < 0.001 vs 94.6% CI: 91.8%–97.4%, SE 1.4%, *p* < 0.001, respectively; Fixed effects).^15,19,21^

The incidence of reported emergency open repair following the failure of the PP device was 3% (95% CI 1.4%–4.7%, SE 0.8%, *p* < 0.001) *I*^2^ = 0% (fixed effects; Table 4).^13–22^ In 381 patients, the incidence of acute limb ischemia after VA-ECMO decannulation was 2.5% (95% CI 0.9%–4.%, SE 0.8%, *p* = 0.002) *I*^2^ = 0% (fixed effects; Table 4).^13–21^ The incidence of infection of the puncture site after decannulation was 1% (95% CI 0%–2%, SE 0.5%, *p* < 0.047) *I*^2^ = 0% (fixed effects) in a total of 385 patients (Table 4).^13–15,17–22^ The incidence of patients with pseudoaneurysm after decannulation was 1.1% (95% CI 0.1%–2.1%, SE 0.5%, *p* < 0.031) *I*^2^ = 0%, evaluated in 403 patients (Table 4).^13–19,21,22^

The incidence of arterial thrombosis was evaluated in 259 patients, with rates between 0% and 6.7%.^13,17–19,21^ Out of 157 patients, there was a single report of an arterio-venous fistula.^13,17–19,22^ However, the cause of this event is dubious as Sun et al. lack evidence to fully determine the reason for this, whether by decannulation or the puncture process. Across 113 patients, the occurrence of hematoma varied from 0% to 10%.^13,18,19,22^ Furthermore, among 189 patients, the frequency of arterial dissection was noted to be between 0% and 6.7%.^17,18,20,21^

Concerning other short-term outcomes in patients that underwent percutaneous VA-ECMO decannulation, available data was sparse but further withdrawn and displayed in Table 4. Four studies reported that hospital stay duration varied from 13.7 to 42.51 days.^13,14,18,20^

### Studies quality

**Figure 3.**
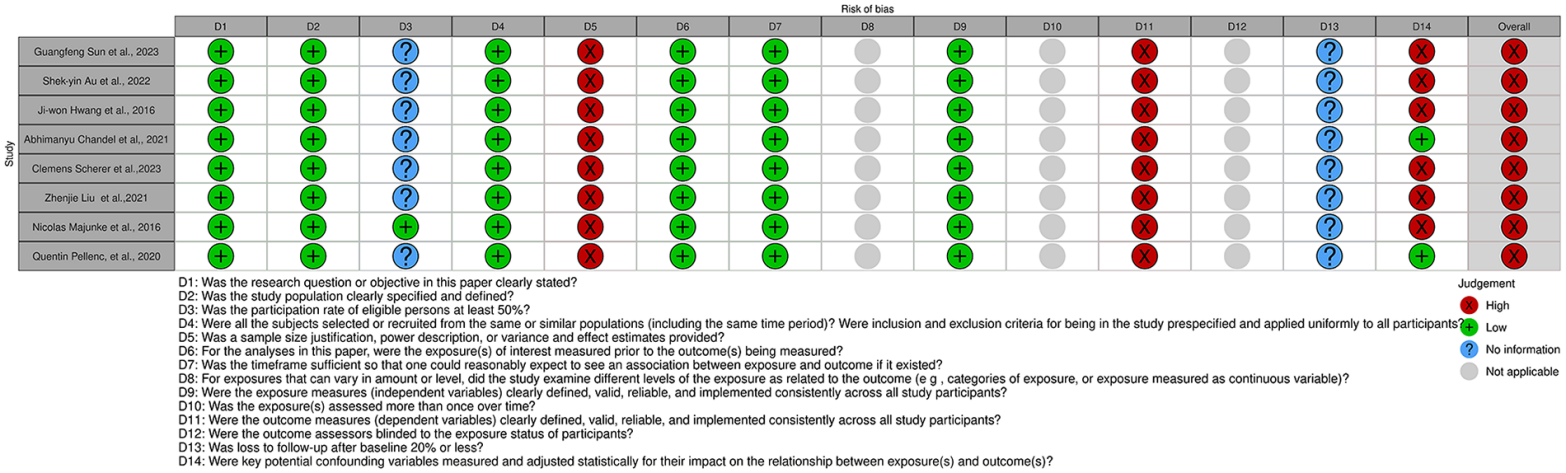
Risk of bias of all cohort studies included in the systematic review, displayed by article.

**Figure 4.**
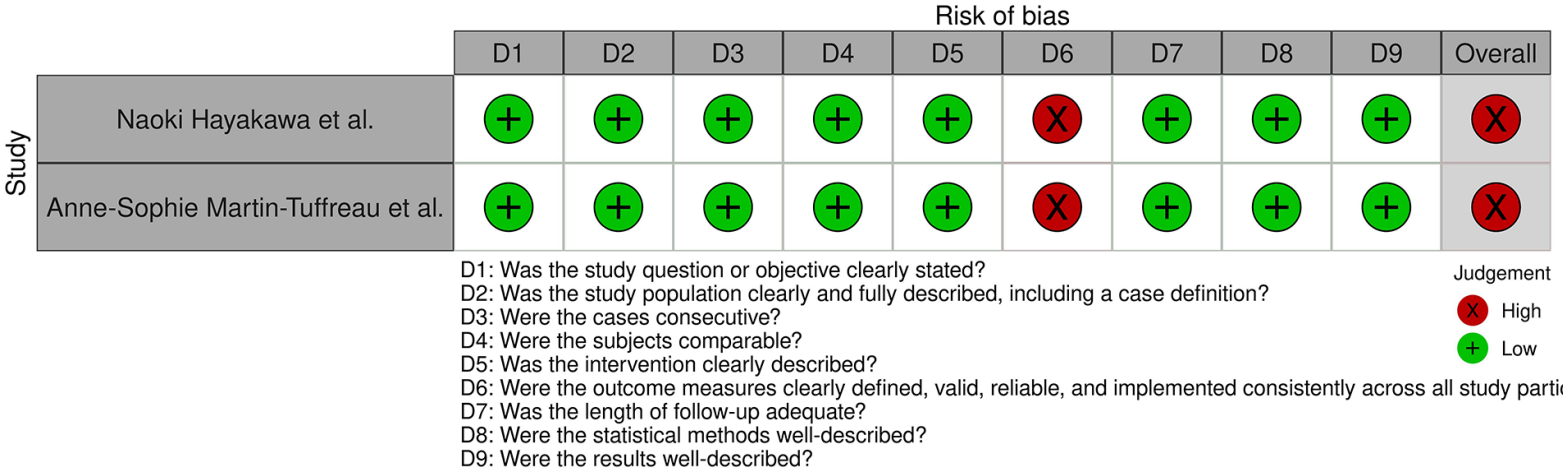
Risk of bias of all Case series studies included in the systematic review, displayed by article.

Figures 3 and 4 and Supplemental Figures 1 and 2 display the risk of bias of the selected articles. Figure 3 displays the risk of bias for each observational cohort individually. Supplemental Figure 1 shows the overall judgment per evaluated item regarding observational cohorts.

All observational cohorts had an overall high risk of bias. The items most frequently associated with a high risk of bias among observational cohorts included sample size justification, outcome measures being consistently implemented across all study participants, and measuring key potential confounding variables.

The specific tool was applied to the case series studies.^22^ Both shared an overall high risk of bias due to outcome measures not being consistently implemented across all study participants. The risk of bias for each case series is individually displayed in Figure 3. The overall judgment per evaluated item regarding case series is shown in Supplemental Figure 2.

The funnel plot showed a slight asymmetry, suggesting a potential low risk of publication bias, which was further supported by Egger’s regression test indicating no statistically significant bias (*p* > 0.05; Supplemental Figure 3).

## Discussion

In this systematic review, the authors focused on the effectiveness of the PP device for VA-ECMO decannulation. This new technique avoids risky and staffing-consuming transport of these patients to the operation theater. The technical success rates closely match those found in other research studies involving the PP system for different endovascular procedures, including endovascular aortic repair (EVAR) and transcatheter aortic valve replacement (TAVR).^23–25^ The study conducted by Liu et al., included in our systematic review, demonstrated this by showing that PP had a similar success rate in EVAR and VA-ECMO patients.^18^

A pre-closure technique using the PP, where the suturing device is deployed after initial access to the vessel,^26^ has been reported to be highly effective for the hemostasis of large-bore sheaths. However, this technique takes time to perform, which may not be compatible with the urgent or emergent setting of the VA-ECMO.^27^ A post-closure technique, where the suture device is deployed after the insertion of the large sheath introducer,^14^ has also been described. However, if the PP fails, the physician must shift to manual compression, and the transition to a surgical approach may be difficult.^14^ Our findings, however, demonstrate that both techniques share a similar efficacy. In this sense, one might argue that a pre-closure technique could be favored in the elective or semi-elective setting, provided appropriate physician’s skills; conversely, in the urgent setting, careful selection of patients based on their risk for closure failure should be sought first and the post-closure technique implemented judiciously.

Anticoagulation is used routinely in patients undergoing VA-ECMO to diminish circuit-associated thrombotic risks.^28^ However, this practice may potentially lead to increased rates of hemorrhagic complications.

Another critical risk factor for vascular complications is the diameter of the CFA. Ahn et al., found that in Korean and female patients, the diameter of the CFA is smaller compared to Western and male patients, respectively.^29^ However, in our findings, these variables did not seem to affect the rate of vascular outcomes. Hwang et al. also described in this population that PP could not control hemostasis if access sites developed an injury at insertion. Other methods, such as surgical removal, may be preferred in these conditions to prevent additional complications.^14^ The PP also features a significant learning curve, underscoring the critical importance of the physician’s experience and skill for its technical success.

Likewise, a shorter hospital stay is often anticipated with percutaneous decannulation techniques compared to surgical cutdown.^30^ These findings strongly suggest that using the PP, as opposed to surgical cutdown, could decrease the length of hospital stays and time spent in the ICU, as the rate of short-term complications is low. However, this could not be conclusively determined since only three studies provided data on ICU stays^14,15,19^ and four studies on hospital stays,^13,14,18,20^ with the reported data being inconsistent in format.

Only four studies reported procedure-related deaths, although no cases were reported in these studies, which is another major indicator of the safety of this procedure.^15,17,20,22^

In most studies, limb ischemia was evaluated through Doppler ultrasound. No studies reported using near-infrared spectroscopy (NIRS) to monitor limb perfusion.^31^ No data about the selection of the limb side were reported either. The selection of the limb side is relevant to preventing limb ischemia in patients with ECMO support.^32^

This study faced many limitations. First, only some articles were eligible for this systematic review, and the majority had a small sample size without sample justifications and power descriptions, which led to low precision of obtained results and might affect external validity. Furthermore, no randomized clinical trials were found in the literature. All studies lack randomization of surgeons’ preferences and experience and patient selection, which most likely influenced the choice in treatment and the findings of the success of the PP. On the other hand, there was severe heterogeneity amongst studies regarding most baseline patient characteristics, study designs, and methodology, prompting a high diversity of indications for VA-ECMO cannulation. Differences between primary studies were so extensive that, in meta-regression, any single variable could be identified, which could account for most heterogeneity. The authors could not perform multivariable meta-regression models because of the insufficient number of primary studies included. Few other short- and long-term outcomes were assessed, such as stroke, which deprived the study of association with different outcomes.

It should be noted that the inconsistency in follow-up periods across studies hampers the clear comprehension of outcome rates, and in some of the studies, the follow-up time might not have been sufficient to truly assess the rate of vascular complications.

However, this study also has strengths. The selection criteria were broad and inclusive, and there weren’t any language restrictions. A respectable number of patients were included, which allowed meaningful quantitative synthesis.

## Conclusion

The evidence presented in our systematic review suggests that PP is a safe and reliable device for achieving hemostasis after percutaneous VA-ECMO decannulation, with a high success rate and low rate of major complications.

Additional research is needed, including larger, multicenter studies with standardized follow-up. A randomized controlled trial (RCT) would be particularly useful to rigorously confirm these findings and accurately determine complication rates. Further studies should also be made to compare the use of PP to other percutaneous techniques available in the market to optimize the VA-ECMO decannulation process.

## Supplemental Material

sj-pdf-1-jva-10.1177_11297298241312753 – Supplemental material for Assessment of safety and effectiveness after percutaneous closure for decannulation of Veno-Arterial Extracorporeal Membrane Oxygenation: A systematic review and meta-analysis Supplemental material, sj-pdf-1-jva-10.1177_11297298241312753 for Assessment of safety and effectiveness after percutaneous closure for decannulation of Veno-Arterial Extracorporeal Membrane Oxygenation: A systematic review and meta-analysis by Tomás Marques Pereira, Diana Martins-Fernandes, Ana Rita Ferreira, Henrique Guedes da Rocha, Mario D’Oria and João Rocha-Neves in The Journal of Vascular Access

sj-pdf-2-jva-10.1177_11297298241312753 – Supplemental material for Assessment of safety and effectiveness after percutaneous closure for decannulation of Veno-Arterial Extracorporeal Membrane Oxygenation: A systematic review and meta-analysis Supplemental material, sj-pdf-2-jva-10.1177_11297298241312753 for Assessment of safety and effectiveness after percutaneous closure for decannulation of Veno-Arterial Extracorporeal Membrane Oxygenation: A systematic review and meta-analysis by Tomás Marques Pereira, Diana Martins-Fernandes, Ana Rita Ferreira, Henrique Guedes da Rocha, Mario D’Oria and João Rocha-Neves in The Journal of Vascular Access

sj-pdf-3-jva-10.1177_11297298241312753 – Supplemental material for Assessment of safety and effectiveness after percutaneous closure for decannulation of Veno-Arterial Extracorporeal Membrane Oxygenation: A systematic review and meta-analysis Supplemental material, sj-pdf-3-jva-10.1177_11297298241312753 for Assessment of safety and effectiveness after percutaneous closure for decannulation of Veno-Arterial Extracorporeal Membrane Oxygenation: A systematic review and meta-analysis by Tomás Marques Pereira, Diana Martins-Fernandes, Ana Rita Ferreira, Henrique Guedes da Rocha, Mario D’Oria and João Rocha-Neves in The Journal of Vascular Access

sj-pdf-4-jva-10.1177_11297298241312753 – Supplemental material for Assessment of safety and effectiveness after percutaneous closure for decannulation of Veno-Arterial Extracorporeal Membrane Oxygenation: A systematic review and meta-analysis Supplemental material, sj-pdf-4-jva-10.1177_11297298241312753 for Assessment of safety and effectiveness after percutaneous closure for decannulation of Veno-Arterial Extracorporeal Membrane Oxygenation: A systematic review and meta-analysis by Tomás Marques Pereira, Diana Martins-Fernandes, Ana Rita Ferreira, Henrique Guedes da Rocha, Mario D’Oria and João Rocha-Neves in The Journal of Vascular Access

sj-pdf-5-jva-10.1177_11297298241312753 – Supplemental material for Assessment of safety and effectiveness after percutaneous closure for decannulation of Veno-Arterial Extracorporeal Membrane Oxygenation: A systematic review and meta-analysis Supplemental material, sj-pdf-5-jva-10.1177_11297298241312753 for Assessment of safety and effectiveness after percutaneous closure for decannulation of Veno-Arterial Extracorporeal Membrane Oxygenation: A systematic review and meta-analysis by Tomás Marques Pereira, Diana Martins-Fernandes, Ana Rita Ferreira, Henrique Guedes da Rocha, Mario D’Oria and João Rocha-Neves in The Journal of Vascular Access
