# Supplementary material for: Assessment of safety and effectiveness after percutaneous closure for decannulation of Veno-Arterial Extracorporeal Membrane Oxygenation: A systematic review and meta-analysis
Source: J Vasc Access. 2025 Jan 29;26(6):1795–805. doi: 10.1177/11297298241312753 (PMC12615847; doi:10.1177/11297298241312753)
Supplement: sj-pdf-1-jva-10.1177_11297298241312753 – Supplemental material for Assessment of safety and effectiveness after percutaneous closure for decannulation of Veno-Arterial Extracorporeal Membrane Oxygenation: A systematic review and meta-analysis [file sj-pdf-1-jva-10.1177_11297298241312753.pdf]

Supplemental Table I: Search Strategy

Databases	Query and Keywords	Articles Found
PubMed	(“ECMO” OR “Extracorporeal membrane oxygenation” OR “Extracorporeal Circulation” OR “ECLS” OR “extracoporeal life-support” OR “venous-arterial” OR “extracorporeal cardiopulmonary resuscitation” OR “ECPR” OR “venoarterial”) AND ("ProGlide" OR “Perclose” OR "vascular closure devices" OR ("vascular" AND "closure"AND "device") OR "vascular closure device" OR “Vascular closure” or “Percutaneous closure” OR “artery closure”)	73
Cochrane	(“ECMO” OR “Extracorporeal membrane oxygenation” OR “Extracorporeal Circulation” OR “ECLS” OR “extracoporeal life-support” OR “venous-arterial” OR “extracorporeal cardiopulmonary resuscitation” OR “ECPR” OR “venoarterial”) AND ("ProGlide" OR “Perclose” OR "vascular closure devices" OR ("vascular" AND "closure"AND "device") OR "vascular closure device" OR “Vascular closure” or “Percutaneous closure” OR “artery closure”)	2
Web of Science	(“ECMO” OR “Extracorporeal membrane oxygenation” OR “Extracorporeal Circulation” OR “ECLS” OR “extracoporeal life-support” OR “venous-arterial” OR “extracorporeal cardiopulmonary resuscitation” OR “ECPR” OR “venoarterial”) AND ("ProGlide" OR “Perclose” OR "vascular closure devices" OR ("vascular" AND "closure"AND "device") OR "vascular closure device" OR “Vascular closure” or “Percutaneous closure” OR “artery closure”)	85
