# Supplementary material for: Assessment of safety and effectiveness after percutaneous closure for decannulation of Veno-Arterial Extracorporeal Membrane Oxygenation: A systematic review and meta-analysis
Source: J Vasc Access. 2025 Jan 29;26(6):1795–805. doi: 10.1177/11297298241312753 (PMC12615847; doi:10.1177/11297298241312753)
Supplement: sj-pdf-2-jva-10.1177_11297298241312753 – Supplemental material for Assessment of safety and effectiveness after percutaneous closure for decannulation of Veno-Arterial Extracorporeal Membrane Oxygenation: A systematic review and meta-analysis [file sj-pdf-2-jva-10.1177_11297298241312753.pdf]

PRISMA 2020 Checklist

Section and Topic	Item #	Checklist item	Location where item is reported
**TITLE**			
Title	1	Identify the report as a systematic review.	"Assessment of safety and effectiveness after percutaneous closure for decannulation of Veno-Arterial Extracorporeal Membrane Oxygenation (VA-ECMO): A Systematic review and meta-analysis"
**ABSTRACT**			
Abstract	2	See the PRISMA 2020 for Abstracts checklist.	"Background: Veno-Arterial Extracorporeal Membrane Oxygenation (VA-ECMO) has emerged as a crucial component of critical care medicine, mainly as a lifesaving intervention for patients experiencing refractory cardiac arrest and respiratory failure.<br>In the past VA-ECMO decannulation was surgical and often associated with a high rate of periprocedural complications, such as surgical site infection, bleeding, and patient mobilization costs. To reduce the rate of these adverse events, many percutaneous techniques, utilizing suture-mediated closing devices have been adopted. One of those devices is the Perclose Proglide® (PP)<br>Objective: The goal of

PRISMA 2020 Checklist

Section and Topic	Item #	Checklist item	Location where item is reported
			this study was to perform a systematic review to evaluate the success and complication rates of the use of PP devices for VA-ECMO decannulation.<br>Methods: A systematic review of the most recent literature was conducted to analyze the outcomes of PP in VA-ECMO decannulation. The Medline, Web of Science and Cochrane database databases were systematically searched up to September 2023. The National Health, Blood and Lung Institute Study quality assessment tools were used.<br>Results: Ten observational studies comprising 418 patients were included in the final analysis. The efficacy of PP in VA-ECMO decannulation was 93.0% [95% CI 90.1–96.0%). In 381 patients, the incidence of acute limb ischemia after VA-ECMO decannulation was 2.5% (95% CI 0.9%-4%), the infection of the puncture site after decannulation was 1% (95% CI 0%-2%) in 385 patients. The incidence of patients

PRISMA 2020 Checklist

Section and Topic	Item #	Checklist item	Location where item is reported
			with pseudoaneurysm after decannulation ranged was 1.1% (95% CI 0.1%-2.1%).<br>Conclusion: This systematic review and meta-analysis demonstrate the safety and efficacy of the PP for achieving haemostasis after VA-ECMO decannulation, with a high success rate and low rate of major complications.”
**INTRODUCTION**			
Rationale	3	Describe the rationale for the review in the context of existing knowledge.	“Veno-Arterial Extracorporeal Membrane Oxygenation (VA-ECMO) has emerged as a crucial component of critical care medicine, mainly as a lifesaving intervention for patients experiencing refractory cardiac arrest and respiratory failure, helping maintain peripheral perfusion as well as restore end-organ function.1 The use of ECMO as a cardiopulmonary support, easiness of implantation, increasing availability with the development of mobile ECMO teams, and a broadening range of indications have contributed to its expansion in the last years.2 Data from the

PRISMA 2020 Checklist

Section and Topic	Item #	Checklist item	Location where item is reported
			Extracorporeal Life Support Organization (ELSO) Registry show a rise in ECMO utilization throughout the years, recording a total number of 16.803 in 2022.3<br><br>VA-ECMO is usually initiated through cannulas placed in the common femoral artery (CFA) and the common femoral vein (CFV), either with an open or percutaneous approach.4 Even though originally VA-ECMO decannulation has been done with surgery, that practice may often be associated with high patient mobilization costs and a high rate of peri-procedural complications, such as bleeding and site infection, with the latter being reported to be as high as 45% in a study done by Paul Haddad et al.5, and patient mobilization costs. Therefore, to reduce the rate of these adverse events, many percutaneous techniques utilizing suture-mediated closing devices have been adopted. One of those devices is the Perclose Proglide® (PP) (Abott laboratories, released 2004, Perclose

PRISMA 2020 Checklist

Section and Topic	Item #	Checklist item	Location where item is reported
			ProGlide™ Suture-Mediated Closure (SMC) System, Illinois, Chicago, USA).65<br>The PP is a device that actively approximates and sutures arteriotomy sites percutaneously. It operates by being inserted over a guidewire until blood flow indicates its correct placement within the lumen, then deploys "feet" to secure against the vessel wall, followed by needle deployment to create a suture loop that closes the arteriotomy upon tightening. Approved for closing 5F to 21F arteriotomy sites as per its Instructions for Use (IFU), the PP requires two devices and a "preclose" technique for sheaths larger than 8F.<br>Despite the abundance of contemporary evidence on the safety and effectiveness of the PP for closure of large-bore arterial access after endovascular aneurysm repair,8 much less is known about its utilization in the field of ECMO.
Objectives	4	Provide an explicit statement of the objective(s) or question(s) the review addresses.	“Therefore, the goal of this study was to perform a systematic

PRISMA 2020 Checklist

Section and Topic	Item #	Checklist item	Location where item is reported
			review to evaluate the safety and effectiveness after use of the PP device for percutaneous VA-ECMO decannulation.”
**METHODS**			
Eligibility criteria	5	Specify the inclusion and exclusion criteria for the review and how studies were grouped for the syntheses.	“Inclusion criteria consisted in all original cohort or experimental studies performed in humans (except for case series under 10 patients), aged 18 years old and over, in which patients underwent percutaneous VA-ECMO arterial cannula removal with the use of PP. No exclusion was made based on the publication language or publication date.”
Information sources	6	Specify all databases, registers, websites, organisations, reference lists and other sources searched or consulted to identify studies. Specify the date when each source was last searched or consulted.	“A systematic search was performed in three databases (PubMed, Web of Science and Cochrane) in September 2023.”
Search strategy	7	Present the full search strategies for all databases, registers and websites, including any filters and limits used.	Supplemental Table
Selection process	8	Specify the methods used to decide whether a study met the inclusion criteria of the review, including how many reviewers screened each record and each report retrieved, whether they worked independently, and if applicable, details of automation tools used in the process.	After duplicates removal, two authors (TP and JRN) independently participated in studies selection; any disagreement was solved by the intervention of a third author (ARF). First, studies were selected by title and abstract, and the remaining ones were eligible for full-text assessment.

PRISMA 2020 Checklist

Section and Topic	Item #	Checklist item	Location where item is reported
			Efforts were made to contact the authors to obtain the full texts that were not publicly available
Data collection process	9	Specify the methods used to collect data from reports, including how many reviewers collected data from each report, whether they worked independently, any processes for obtaining or confirming data from study investigators, and if applicable, details of automation tools used in the process.	"Data from the included studies were independently extracted by two authors (TP and JRN). Data was extracted using a .xls purposely built form on the year of publication, continent, recruitment center, study design, recruitment time, number of participants undergoing percutaneous VA-ECMO removal using PP, participants' age and gender distribution, frequency of cardiovascular comorbidities, platelet number before decannulation and use of antiplatelet and anticoagulation medication. In addition, data related to technical success of PP, as well as incidence of 30 days/short-term technical success and adverse events after VA-ECMO decannulation was also retrieved."
Data items	10a	List and define all outcomes for which data were sought. Specify whether all results that were compatible with each outcome domain in each study were sought (e.g. for all measures, time points, analyses), and if not, the methods used to decide which results to collect.	"Data from the included studies were independently extracted by two authors (TP and JRN). Data was extracted

Section and Topic	Item #	Checklist item	Location where item is reported
			using a .xls purposely built form on the year of publication, continent, recruitment center, study design, recruitment time, number of participants undergoing percutaneous VA-ECMO removal using PP, participants' age and gender distribution, frequency of cardiovascular comorbidities, platelet number before decannulation, use of antiplatelet and anticoagulation medication. In addition, data was gathered on indication for VA-ECMO, puncture site, cannula size, number of PP devices used per patient, use of preclose technique, use of fluoroscopic guidance, time spent in ecmo and duration of ICU and hospital stay. Data related to technical success of PP, as well as incidence of 30 days/short-term technical success and adverse events after VA-ECMO decannulation, such as acute limb ischemia, arterial thrombosis, pseudoaneurysm, wound infection, arterious-venous fistula, hematoma, arterial dissection and procedure related-

Section and Topic	Item #	Checklist item	Location where item is reported
			death and incidence of open repair following PP device failure was also retrieved.”
	10b	List and define all other variables for which data were sought (e.g. participant and intervention characteristics, funding sources). Describe any assumptions made about any missing or unclear information.	Table I, II and III
Study risk of bias assessment	11	Specify the methods used to assess risk of bias in the included studies, including details of the tool(s) used, how many reviewers assessed each study and whether they worked independently, and if applicable, details of automation tools used in the process.	“Concerning qualitative assessment, the National Heart, Lung, and Blood Institute (NHLBI) Study Quality Assessment Tool was used for observational cohort and cross-sectional studies (2013).11 This assessment was independently performed by two authors (TP and JRN), and when disagreements were observed, decisions were made by mutual consensus after a third-party review (ARF).
Effect measures	12	Specify for each outcome the effect measure(s) (e.g. risk ratio, mean difference) used in the synthesis or presentation of results.	“A random-effects meta-analysis (using the restricted maximum likelihood method) of log-transformed proportions to calculate the meta-analytical pooled incidence of an efficient and safe percutaneous closure among participants was performed. Pooled estimates and 95% confidence intervals (95%CI) were back transformed into their original scale to simplify their interpretation.

Section and Topic	Item #	Checklist item	Location where item is reported
			Heterogeneity was assessed using the Q-Cochran p-value and the I2 statistic – a p-value<0.10 and an I2≥50% were considered to represent substantial heterogeneity. Sources of heterogeneity were assessed by leave-one-out sensitivity analysis. Assessed covariates included the publication year, participants' mean age, percentage of male participants, percentage of patients with arterial atherosclerotic risk factors, percentage of patients using antiplatelet and anticoagulant drugs.<br>All statistical analysis were performed using Open Meta® (MetaMorph, Inc)."
Synthesis methods	13a	Describe the processes used to decide which studies were eligible for each synthesis (e.g. tabulating the study intervention characteristics and comparing against the planned groups for each synthesis (item #5)).	"Data from the included studies were independently extracted by two authors (TP and JRN). Data was extracted using a .xls purposely built form on the year of publication, continent, recruitment center, study design, recruitment time, number of participants undergoing percutaneous VA-ECMO removal using PP, participants' age and gender

Section and Topic	Item #	Checklist item	Location where item is reported
			distribution, frequency of cardiovascular comorbidities, platelet number before decannulation and use of antiplatelet and anticoagulation medication. In addition, data related to technical success of PP, as well as incidence of 30 days/short-term technical success and adverse events after VA-ECMO decannulation was also retrieved.”
	13b	Describe any methods required to prepare the data for presentation or synthesis, such as handling of missing summary statistics, or data conversions.	No data synthesis was performed.
	13c	Describe any methods used to tabulate or visually display results of individual studies and syntheses.	No data synthesis was performed.
	13d	Describe any methods used to synthesize results and provide a rationale for the choice(s). If meta-analysis was performed, describe the model(s), method(s) to identify the presence and extent of statistical heterogeneity, and software package(s) used.	“A random-effects meta-analysis (using the restricted maximum likelihood method) of log-transformed proportions to calculate the meta-analytical pooled incidence of an efficient and safe percutaneous closure among participants was performed. Pooled estimates and 95% confidence intervals (95%CI) were back transformed into their original scale to simplify their interpretation. Heterogeneity was assessed using the Q-Cochran p-value and the I2 statistic – a p-

Section and Topic	Item #	Checklist item	Location where item is reported
			value<0.10 and an I2≥50% were considered to represent substantial heterogeneity. Sources of heterogeneity were assessed by leave-one-out sensitivity analysis. Assessed covariates included the publication year, participants' mean age, percentage of male participants, percentage of patients with arterial atherosclerotic risk factors, percentage of patients using antiplatelet and anticoagulant drugs.<br>All statistical analysis were performed using Open Meta® (MetaMorph, Inc)."
	13e	Describe any methods used to explore possible causes of heterogeneity among study results (e.g. subgroup analysis, meta-regression).	Nothing to report.
	13f	Describe any sensitivity analyses conducted to assess robustness of the synthesized results.	"Heterogeneity was assessed using the Q-Cochran p-value and the I2 statistic – a p-value<0.10 and an I2≥50% were considered to represent substantial heterogeneity. Sources of heterogeneity were assessed by leave-one-out sensitivity analysis."
Reporting bias assessment	14	Describe any methods used to assess risk of bias due to missing results in a synthesis (arising from reporting biases).	"Concerning qualitative assessment, the National Heart, Lung, and Blood Institute (NHLBI) Study Quality Assessment Tool was

PRISMA 2020 Checklist

Section and Topic	Item #	Checklist item	Location where item is reported
			used for observational cohort and case series studies (2013).11 This assessment was independently performed by two authors (TP and JRN), and when disagreements were observed, decisions were made by mutual consensus after a third-party review (ARF).”
Certainty assessment	15	Describe any methods used to assess certainty (or confidence) in the body of evidence for an outcome.	Not possible.
**RESULTS**			
Study selection	16a	Describe the results of the search and selection process, from the number of records identified in the search to the number of studies included in the review, ideally using a flow diagram.	Figure 1
	16b	Cite studies that might appear to meet the inclusion criteria, but which were excluded, and explain why they were excluded.	“After the database search and duplicate exclusion, a total of 160 studies were screened. Upon selection by title and abstract, 85 studies were excluded. 13 studies were eligible for full-text assessment, and, during this process, no studies were excluded or not retrieved (Figure 1). Comprehensive reasons for exclusion upon full-text assessment were: repeated date base (N=3), and absence of full-text even after contacting the respective author (N=1). Thus, a total of 10 published articles were included in this systematic review and

PRISMA 2020 Checklist

Section and Topic	Item #	Checklist item	Location where item is reported
			included in the meta-analysis (Table I)."
Study characteristics	17	Cite each included study and present its characteristics.	"This review includes 8 observational cohort studies and 2 case series . Overall, 9 of those studies were retrospective.12-20 and 1 was prospective.21 Characteristics of the studies and case series can be found in table I. The included publications were performed in 7 different countries within 3 continents (1 from North America, 4 from European countries, and 5 from Asia). A total of 418 patients were assessed, from a minimum of 15 up to a maximum of 106 patients per study. The mean participants' age was 56.2 years old. The percentage of male participants was 56.0% (n=248). Demographics and comorbidities of the populations included in the studies were gathered and are available in Table II
Risk of bias in studies	18	Present assessments of risk of bias for each included study.	Figures 2 and 4
Results of individual studies	19	For all outcomes, present, for each study: (a) summary statistics for each group (where appropriate) and (b) an effect estimate and its precision (e.g. confidence/credible interval), ideally using structured tables or plots.	"In 418 patients, the meta-analytical technical success rate of PP in VA-ECMO decannulation was 93.0% [95% CI 90.1–96.0%), Standard error

Section and Topic	Item #	Checklist item	Location where item is reported
			(SE) 1.2%, p<0.001] I2 =19.8% (Fixed effects). In all results of leave-one-out sensitivity, the overall estimate remained stable across all iterations.<br>Additionally, subgroup analysis regarding the use of pre-closure technique revealed an efficiency similar to the post-removal technique (91.7% CI: 87.7%-95.9%, SE 2.1%, p<0.001 vs. 94.6% CI: 91.8%-97.4%, SE: 1.4%, p<0.001, respectively) (Fixed effects).14,18,20<br>The incidence of reported emergency open repair following failure of the PP device was 3% (95% CI 1.4%-4.7%, SE 0.8%, p<0.001) I2=0% (fixed effects) (Table IV).12-21. In 381 patients, the incidence of acute limb ischemia after VA-ECMO decannulation was 2.5% (95% CI 0.9%-4. %, SE 0.8%, p=0.002) I2=0% (fixed effects) (Table IV).12-20 Incidence of infection of the puncture site after decannulation was 1% (95% CI 0%-2%, SE 0.5%, p<0.047) I2=0% (fixed effects), in a total of 385 patients (Table IV).12-14,16-21 The incidence of patients with pseudoaneurysm

Section and Topic	Item #	Checklist item	Location where item is reported
			after decannulation was 1.1% (95% CI 0.1%-2.1%, SE 0.5%, p<0.031) I2=0%, evaluated in 403 patients (Table IV).12-18,20,21<br>The incidence of arterial thrombosis was evaluated in 259 patients, with rates observed to vary between 0% and 6.7%.12,16-18,20 Out of 157 patients, there was a single report of an arterio-venous fistula.12,16-18,21 The cause of this event, however, is dubious as Guangfeng Sun et al. lack evidence to fully determine the cause for this, whether by decannulation or the puncture process. Across 113 patients, the occurrence of hematoma varied from 0% to 10%.12,17,18,21 Furthermore, among 189 patients, the frequency of arterial dissection was noted to be between 0% and 6.7%.16,17,19,20<br><br>Concerning other short-term outcomes in patients that underwent percutaneous VA-ECMO decannulation, available data was sparse but further withdrawn and displayed in Table IV.

Section and Topic	Item #	Checklist item	Location where item is reported
			Hospital stay duration was reported by four studies, varying from 13.7 to 42.51 days. 12,13,17,19”
Results of syntheses	20a	For each synthesis, briefly summarise the characteristics and risk of bias among contributing studies.	Figures 2, 3, 4 and 5
	20b	Present results of all statistical syntheses conducted. If meta-analysis was done, present for each the summary estimate and its precision (e.g. confidence/credible interval) and measures of statistical heterogeneity. If comparing groups, describe the direction of the effect.	“In 418 patients, the meta-analytical technical success rate of PP in VA-ECMO decannulation was 93.0% [95% CI 90.1–96.0%), Standard error (SE) 1.2%, p<0.001] I2 =19.8% (Fixed effects). In all results of leave-one-out sensitivity, the overall estimate remained stable across all iterations.<br>Additionally, subgroup analysis regarding the use of pre-closure technique revealed an efficiency similar to the post-removal technique (91.7% CI: 87.7%-95.9%, SE 2.1%, p<0.001 vs. 94.6% CI: 91.8%-97.4%, SE: 1.4%, p<0.001, respectively) (Fixed effects).14,18,20<br>The incidence of reported emergency open repair following failure of the PP device was 3% (95% CI 1.4%-4.7%, SE 0.8%, p<0.001) I2=0% (fixed effects) (Table IV).12-21. In 381 patients, the incidence of acute limb ischemia after VA-ECMO decannulation was 2.5% (95% CI

Section and Topic	Item #	Checklist item	Location where item is reported
			0.9%-4. %, SE 0.8%, p=0.002) I2=0% (fixed effects) (Table IV).12-20 Incidence of infection of the puncture site after decannulation was 1% (95% CI 0%-2%, SE 0.5%, p<0.047) I2=0% (fixed effects), in a total of 385 patients (Table IV).12-14,16-21 The incidence of patients with pseudoaneurysm after decannulation was 1.1% (95% CI 0.1%-2.1%, SE 0.5%, p<0.031) I2=0%, evaluated in 403 patients (Table IV).12-18,20,21<br>The incidence of arterial thrombosis was evaluated in 259 patients, with rates observed to vary between 0% and 6.7%.12,16-18,20 Out of 157 patients, there was a single report of an arterio-venous fistula.12,16-18,21 The cause of this event, however, is dubious as Guangfeng Sun et al. lack evidence to fully determine the cause for this, whether by decannulation or the puncture process. Across 113 patients, the occurrence of hematoma varied from 0% to 10%.12,17,18,21 Furthermore, among 189 patients, the frequency of arterial

Section and Topic	Item #	Checklist item	Location where item is reported
			dissection was noted to be between 0% and 6.7%.16,17,19,20<br><br>Concerning other short-term outcomes in patients that underwent percutaneous VA-ECMO decannulation, available data was sparse but further withdrawn and displayed in Table IV. Hospital stay duration was reported by four studies, varying from 13.7 to 42.51 days. 12,13,17,19"
	20c	Present results of all investigations of possible causes of heterogeneity among study results.	In 418 patients, the meta-analytical technical success rate of PP in VA-ECMO decannulation was 93.0% [95% CI 90.1–96.0%), Standard error (SE) 1.2%, p<0.001] I2 =19.8% (Fixed effects). In all results of leave-one-out sensitivity, the overall estimate remained stable across all iterations.<br><br>Additionally, subgroup analysis regarding the use of pre-closure technique revealed an efficiency similar to the post-removal technique (91.7% CI: 87.7%-95.9%, SE 2.1%, p<0.001 vs. 94.6% CI: 91.8%-97.4%, SE: 1.4%, p<0.001, respectively) (Fixed effects).14,18,20<br><br>The incidence of

Section and Topic	Item #	Checklist item	Location where item is reported
			reported emergency open repair following failure of the PP device was 3% (95% CI 1.4%-4.7%, SE 0.8%, p<0.001) I2=0% (fixed effects) (Table IV).12-21. In 381 patients, the incidence of acute limb ischemia after VA-ECMO decannulation was 2.5% (95% CI 0.9%-4. %, SE 0.8%, p=0.002) I2=0% (fixed effects) (Table IV).12-20 Incidence of infection of the puncture site after decannulation was 1% (95% CI 0%-2%, SE 0.5%, p<0.047) I2=0% (fixed effects), in a total of 385 patients (Table IV).12-14,16-21 The incidence of patients with pseudoaneurysm after decannulation was 1.1% (95% CI 0.1%-2.1%, SE 0.5%, p<0.031) I2=0%, evaluated in 403 patients (Table IV).12-18,20,21<br>The incidence of arterial thrombosis was evaluated in 259 patients, with rates observed to vary between 0% and 6.7%.12,16-18,20 Out of 157 patients, there was a single report of an arterio-venous fistula.12,16-18,21 The cause of this event, however, is dubious as Guangfeng Sun et al.

Section and Topic	Item #	Checklist item	Location where item is reported
			lack evidence to fully determine the cause for this, whether by decannulation or the puncture process. Across 113 patients, the occurrence of hematoma varied from 0% to 10%.12,17,18,21 Furthermore, among 189 patients, the frequency of arterial dissection was noted to be between 0% and 6.7%.16,17,19,20<br><br>Concerning other short-term outcomes in patients that underwent percutaneous VA-ECMO decannulation, available data was sparse but further withdrawn and displayed in Table IV. Hospital stay duration was reported by four studies, varying from 13.7 to 42.51 days. 12,13,17,19
	20d	Present results of all sensitivity analyses conducted to assess the robustness of the synthesized results.	"In 418 patients, the meta-analytical technical success rate of PP in VA-ECMO decannulation was 93.0% [95% CI 90.1–96.0%), Standard error (SE) 1.2%, p<0.001] I2 =19.8% (Fixed effects). In all results of leave-one-out sensitivity, the overall estimate remained stable across all iterations.<br><br>Additionally, subgroup analysis regarding the

PRISMA 2020 Checklist

Section and Topic	Item #	Checklist item	Location where item is reported
			use of pre-closure technique revealed an efficiency similar to the post-removal technique (91.7% CI: 87.7%-95.9%, SE 2.1%, p<0.001 vs. 94.6% CI: 91.8%-97.4%, SE: 1.4%, p<0.001, respectively) (Fixed effects).14,18,20<br>The incidence of reported emergency open repair following failure of the PP device was 3% (95% CI 1.4%-4.7%, SE 0.8%, p<0.001) I2=0% (fixed effects) (Table IV).12-21. In 381 patients, the incidence of acute limb ischemia after VA-ECMO decannulation was 2.5% (95% CI 0.9%-4. %, SE 0.8%, p=0.002) I2=0% (fixed effects) (Table IV).12-20 Incidence of infection of the puncture site after decannulation was 1% (95% CI 0%-2%, SE 0.5%, p<0.047) I2=0% (fixed effects), in a total of 385 patients (Table IV).12-14,16-21 The incidence of patients with pseudoaneurysm after decannulation was 1.1% (95% CI 0.1%-2.1%, SE 0.5%, p<0.031) I2=0%, evaluated in 403 patients (Table IV).12-18,20,21<br>The incidence of arterial thrombosis was

PRISMA 2020 Checklist

Section and Topic	Item #	Checklist item	Location where item is reported
			evaluated in 259 patients, with rates observed to vary between 0% and 6.7%.12,16-18,20 Out of 157 patients, there was a single report of an arterio-venous fistula.12,16-18,21 The cause of this event, however, is dubious as Guangfeng Sun et al. lack evidence to fully determine the cause for this, whether by decannulation or the puncture process. Across 113 patients, the occurrence of hematoma varied from 0% to 10%.12,17,18,21 Furthermore, among 189 patients, the frequency of arterial dissection was noted to be between 0% and 6.7%.16,17,19,20<br><br>Concerning other short-term outcomes in patients that underwent percutaneous VA-ECMO decannulation, available data was sparse but further withdrawn and displayed in Table IV. Hospital stay duration was reported by four studies, varying from 13.7 to 42.51 days. 12,13,17,19”
Reporting biases	21	Present assessments of risk of bias due to missing results (arising from reporting biases) for each synthesis assessed.	Nothing to report.
Certainty of evidence	22	Present assessments of certainty (or confidence) in the body of evidence for each outcome assessed.	“In 418 patients, the meta-analytical technical success rate

Section and Topic	Item #	Checklist item	Location where item is reported
			of PP in VA-ECMO decannulation was 93.0% [95% CI 90.1–96.0%), Standard error (SE) 1.2%, p<0.001] I2 =19.8% (Fixed effects). In all results of leave-one-out sensitivity, the overall estimate remained stable across all iterations.<br><br>Additionally, subgroup analysis regarding the use of pre-closure technique revealed an efficiency similar to the post-removal technique (91.7% CI: 87.7%-95.9%, SE 2.1%, p<0.001 vs. 94.6% CI: 91.8%-97.4%, SE: 1.4%, p<0.001, respectively) (Fixed effects).14,18,20<br><br>The incidence of reported emergency open repair following failure of the PP device was 3% (95% CI 1.4%-4.7%, SE 0.8%, p<0.001) I2=0% (fixed effects) (Table IV).12-21. In 381 patients, the incidence of acute limb ischemia after VA-ECMO decannulation was 2.5% (95% CI 0.9%-4. %, SE 0.8%, p=0.002) I2=0% (fixed effects) (Table IV).12-20 Incidence of infection of the puncture site after decannulation was 1% (95% CI 0%-2%, SE 0.5%, p<0.047) I2=0% (fixed effects), in a total

Section and Topic	Item #	Checklist item	Location where item is reported
			of 385 patients (Table IV).12-14,16-21 The incidence of patients with pseudoaneurysm after decannulation was 1.1% (95% CI 0.1%-2.1%, SE 0.5%, p<0.031) I2=0%, evaluated in 403 patients (Table IV).12-18,20,21<br><br>The incidence of arterial thrombosis was evaluated in 259 patients, with rates observed to vary between 0% and 6.7%.12,16-18,20 Out of 157 patients, there was a single report of an arterio-venous fistula.12,16-18,21 The cause of this event, however, is dubious as Guangfeng Sun et al. lack evidence to fully determine the cause for this, whether by decannulation or the puncture process. Across 113 patients, the occurrence of hematoma varied from 0% to 10%.12,17,18,21 Furthermore, among 189 patients, the frequency of arterial dissection was noted to be between 0% and 6.7%16,17,19,20<br><br>Concerning other short-term outcomes in patients that underwent percutaneous VA-ECMO decannulation,

PRISMA 2020 Checklist

Section and Topic	Item #	Checklist item	Location where item is reported
			available data was sparse but further withdrawn and displayed in Table IV. Hospital stay duration was reported by four studies, varying from 13.7 to 42.51 days. 12,13,17,19”
**DISCUSSION**			
Discussion	23a	Provide a general interpretation of the results in the context of other evidence.	“In this systematic review, the authors focused on the effectiveness of the PP device for VA-ECMO decannulation, a new technique that avoids the risky ant man-power consuming transport of these patients to the operation theatre. The rates of technical success, closely match those found in other research studies, involving the PP system for different endovascular procedures, including endovascular aortic repair (EVAR) and transcatheter aortic valve replacement (TAVR).22-24 The study conducted by Zhenjie Liu et al., included in our systematic review, demonstrated this, by showing that the use of PP had a similar success rate in EVAR and VA-ECMO patients.17<br>A pre-closure

PRISMA 2020 Checklist

Section and Topic	Item #	Checklist item	Location where item is reported
			technique, where the suture device is deployed after initial access of the vessel and artery prior to the insertion of a large sheath introducer,25 using the PP has been reported to be highly effective for haemostasis of large-bore sheaths. Despite this, as VA-ECMO is done in an urgent or emergent setting, this technique is difficult to perform.26 A post-closure technique, where the suture device is deployed after the insertion of the large sheat introducer,13 has also been described. However, if the PP fails, the physician must shift to manual compression, and the transition to surgical approach may be difficult.13 Our findings however demonstrate that both techniques share a similar efficacy. In this sense, one might argue that a pre-closure technique could be favoured in the elective or semi-elective setting, provided appropriate physician's skills; conversely, in the urgent setting, careful selection of patients based on their risk for closure failure, should

Section and Topic	Item #	Checklist item	Location where item is reported
			be sought first and the post-closure technique implemented judiciously.<br><br>It is worth nothing that use of anticoagulation in patients undergoing VA-ECMO is common and is done to diminish circuit-associated thrombotic risks.27 However, this practice may potentially lead to increased rates of haemorrhagic complications.<br><br>Another very important risk factor for vascular complications is the diameter of the CFA. Ho-Young Ahn et al, found that in Korean and female patients the diameter of the CFA is smaller compared to western and male patients, respectively.28 However, in our findings, these variables did not seem to affect the rate of vascular outcomes. Ji-won Hwang et al also described in this population that if access sites developed an injury at insertion, PP was not able to control haemostasis. In these conditions, to prevent additional complications, other methods such as surgical removal, may

Section and Topic	Item #	Checklist item	Location where item is reported
			be preferred.13<br>The PP also features a significant learning curve, underscoring the critical importance of the physician's experience and skill for its technical success.<br>Likewise, a shorter hospital stay is often anticipated with percutaneous decannulation techniques when compared to surgical cutdown.29 These findings strongly suggest that using the PP, as opposed to surgical cutdown, could decrease both the length of hospital stays and time spent in the ICU, as the rate of short-term complications is low. However, this could not be conclusively determined since only three studies provided data on ICU instay,13,14,18 and four studies on hospital instay,12,13,17,19 with the reported data being inconsistent in format.<br>Only 4 studies, reported procedure-related death and, from those studies, no cases were reported, which is another major indicator of the safety of this procedure.14,16,19,21"
	23b	Discuss any limitations of the evidence included in the review.	"This study faced many

PRISMA 2020 Checklist

Section and Topic	Item #	Checklist item	Location where item is reported
			limitations. First, few articles were eligible for this systematic review, and the majority of those had a small sample size without sample justifications and power descriptions, which led to low precision of obtained results and consequently might affect external validity. Furthermore, no randomized clinical trials were found in the literature. All studies lack randomization surgeons´ preference and experience and patient selection, that most likely influenced the choice in treatment and influenced the findings of the success of the PP. On the other hand, there was severe heterogeneity amongst studies regarding most baseline patient characteristics, study designs, and methodology, prompting a high diversity of indications for VA-ECMO cannulation. In fact, differences between primary studies were so extensive that, in meta-regression, any single variable could be identified, which could possibly account for most heterogeneity. The authors were not able to perform

Section and Topic	Item #	Checklist item	Location where item is reported
			multivariable meta-regression models on account of the insufficient number of included primary studies. Few other short- and long-term outcomes were assessed, such as stroke, which deprived the study association with other outcomes.<br><br>It should be noted that the inconsistency in follow-up periods across studies hamper the clear comprehension of outcome rates and in some of the studies the follow-up time might have not been sufficient to truly access the rate of vascular complications.”
	23c	Discuss any limitations of the review processes used.	“This study faced many limitations. First, few articles were eligible for this systematic review, and the majority of those had a small sample size without sample justifications and power descriptions, which led to low precision of obtained results and consequently might affect external validity. Furthermore, no randomized clinical trials were found in the literature. All studies lack randomization surgeons´ preference and experience and

PRISMA 2020 Checklist

Section and Topic	Item #	Checklist item	Location where item is reported
			patient selection, that most likely influenced the choice in treatment and influenced the findings of the success of the PP. On the other hand, there was severe heterogeneity amongst studies regarding most baseline patient characteristics, study designs, and methodology, prompting a high diversity of indications for VA-ECMO cannulation. In fact, differences between primary studies were so extensive that, in meta-regression, any single variable could be identified, which could possibly account for most heterogeneity. The authors were not able to perform multivariable meta-regression models on account of the insufficient number of included primary studies. Few other short- and long-term outcomes were assessed, such as stroke, which deprived the study association with other outcomes.<br><br>It should be noted that the inconsistency in follow-up periods across studies hamper the clear comprehension of outcome rates and in some of the studies the

PRISMA 2020 Checklist

Section and Topic	Item #	Checklist item	Location where item is reported
			follow-up time might have not been sufficient to truly access the rate of vascular complications.”
	23d	Discuss implications of the results for practice, policy, and future research.	“The evidence presented in our systematic review suggests that PP is a safe and reliable device for achieving haemostasis after percutaneous VA-ECMO decannulation, with a high success rate and low rate of major complications.<br>Additional research should be made, as there is a need for larger, multicenter studies that have a standardized follow-up to confirm these findings and determine complication rates rigorously. Further studies should be made as well to compare the use of PP to other percutaneous techniques available in the market to optimize the VA-ECMO decannulation process.”
**OTHER INFORMATION**			
Registration and protocol	24a	Provide registration information for the review, including register name and registration number, or state that the review was not registered.	“The review protocol has been registered on Prospero (reference: CRD42023478774).”
	24b	Indicate where the review protocol can be accessed, or state that a protocol was not prepared.	Fully available.
	24c	Describe and explain any amendments to information provided at registration or in the protocol.	Nothing to report.
Support	25	Describe sources of financial or non-financial support for the review, and the role of the funders or sponsors in the review.	Nothing to report.

PRISMA 2020 Checklist

Section and Topic	Item #	Checklist item	Location where item is reported
Competing interests	26	Declare any competing interests of review authors.	“Financial Support: This research did not receive any specific grant from funding agencies in the public, commercial, or not-for-profit sectors.<br>Conflicts of interest: The Authors declare that there is no conflict of interest.”
Availability of data, code and other materials	27	Report which of the following are publicly available and where they can be found: template data collection forms; data extracted from included studies; data used for all analyses; analytic code; any other materials used in the review.	Supplemental Table I: Search Strategy

From: Page MJ, McKenzie JE, Bossuyt PM, Boutron I, Hoffmann TC, Mulrow CD, et al. The PRISMA 2020 statement: an updated guideline for reporting systematic reviews. BMJ 2021;372:n71. doi: 10.1136/bmj.n71

For more information, visit: http://www.prisma-statement.org/
