# Supplementary figures and images for: Assessment of safety and effectiveness after percutaneous closure for decannulation of Veno-Arterial Extracorporeal Membrane Oxygenation: A systematic review and meta-analysis
Source: J Vasc Access. 2025 Jan 29;26(6):1795–805. doi: 10.1177/11297298241312753 (PMC12615847; doi:10.1177/11297298241312753)

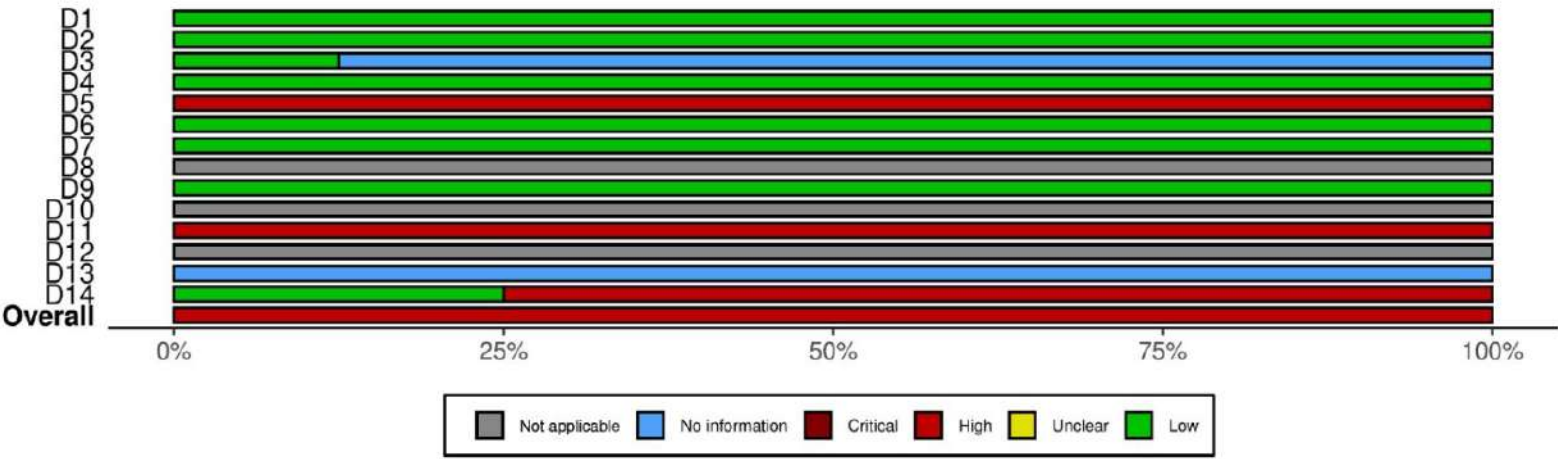

Supplement: sj-pdf-3-jva-10.1177_11297298241312753 – Supplemental material for Assessment of safety and effectiveness after percutaneous closure for decannulation of Veno-Arterial Extracorporeal Membrane Oxygenation: A systematic review and meta-analysis [file sj-pdf-3-jva-10.1177_11297298241312753.pdf]

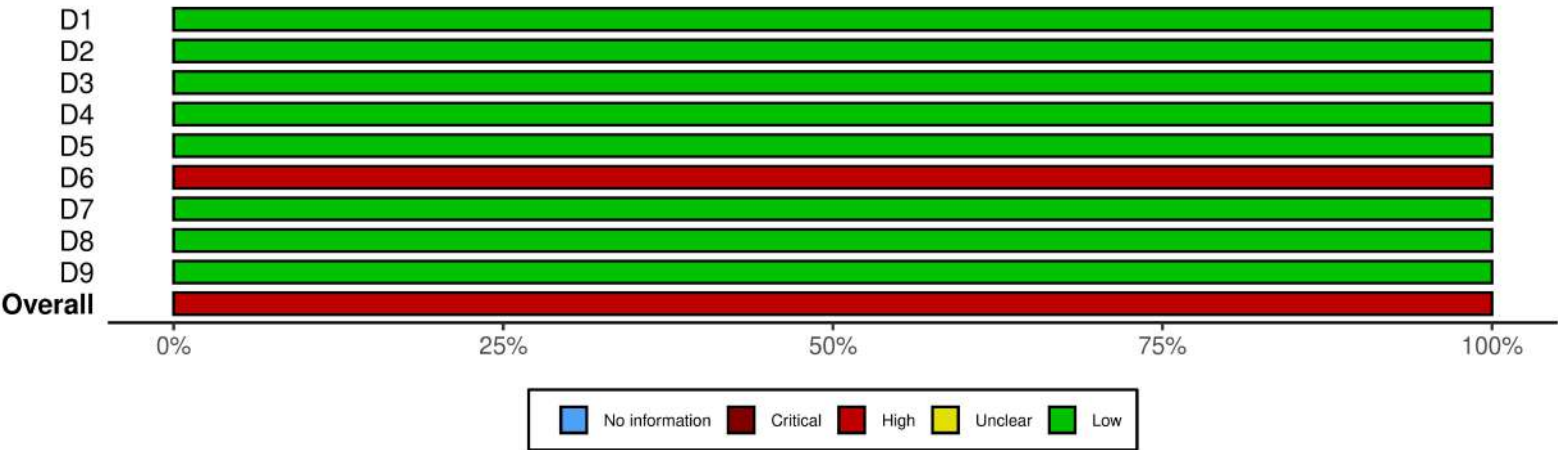

Supplement: sj-pdf-4-jva-10.1177_11297298241312753 – Supplemental material for Assessment of safety and effectiveness after percutaneous closure for decannulation of Veno-Arterial Extracorporeal Membrane Oxygenation: A systematic review and meta-analysis [file sj-pdf-4-jva-10.1177_11297298241312753.pdf]

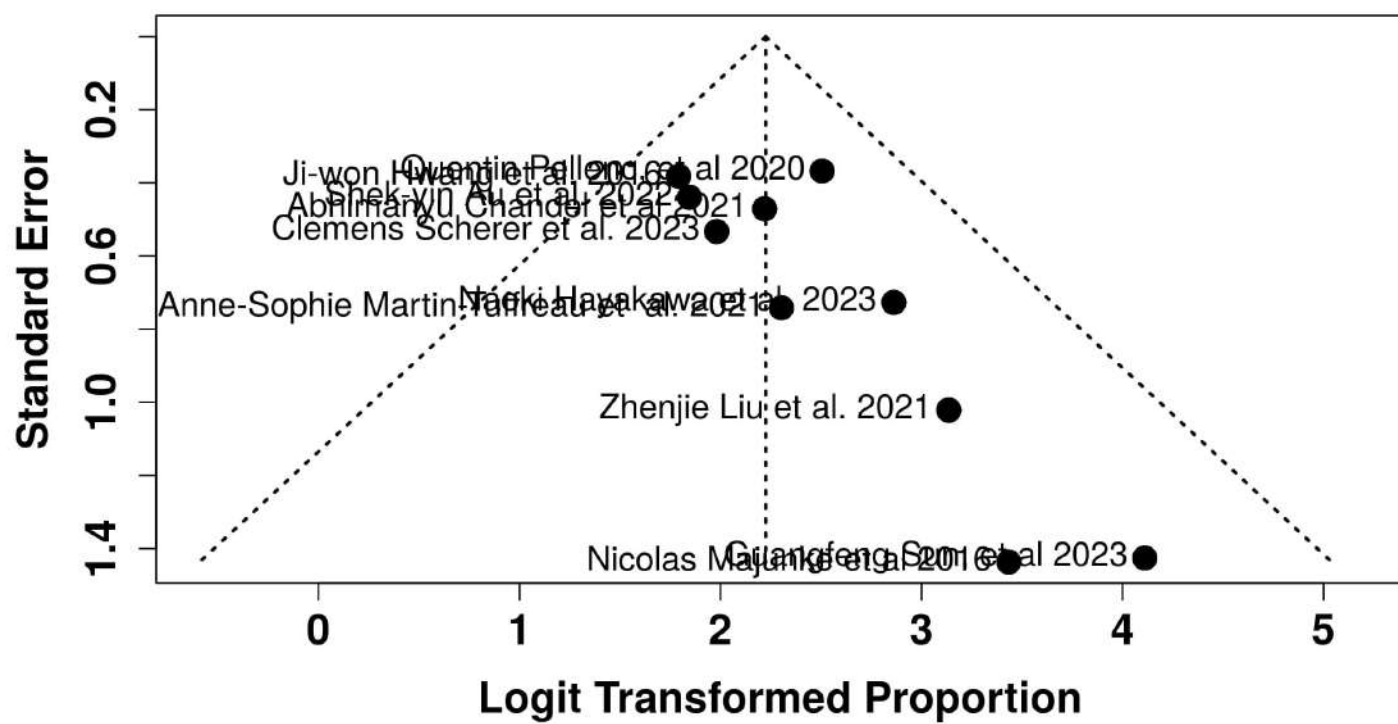

Supplement: sj-pdf-5-jva-10.1177_11297298241312753 – Supplemental material for Assessment of safety and effectiveness after percutaneous closure for decannulation of Veno-Arterial Extracorporeal Membrane Oxygenation: A systematic review and meta-analysis [file sj-pdf-5-jva-10.1177_11297298241312753.pdf]
